# Correction: Mutsuzaki, H., *et al*. Improved Bonding of Partially Osteomyelitic Bone to Titanium Pins Owing to Biomimetic Coating of Apatite. *Int. J. Mol. Sci.* 2013, *14*, 24366–24379

**DOI:** 10.3390/ijms15069789

**Published:** 2014-05-30

**Authors:** Hirotaka Mutsuzaki, Yu Sogo, Ayako Oyane, Atsuo Ito

**Affiliations:** 1 Department of Orthopaedic Surgery, Ibaraki Prefectural University of Health Sciences, 4669-2 Ami Ami-machi, Inashiki-gun, Ibaraki 300-0394, Japan; E-Mail: mutsuzaki@ipu.ac.jp; 2 Human Technology Research Institute, National Institute of Advanced Industrial Science and Technology (AIST), Central 6, 1-1-1 Higashi, Tsukuba-shi, Ibaraki 305-8566, Japan; E-Mail: atsuo-ito@aist.go.jp; 3 Nanosystem Research Institute, National Institute of Advanced Industrial Science and Technology (AIST), Central 4, 1-1-1 Higashi, Tsukuba-shi, Ibaraki 305-8562, Japan; E-Mail: a-oyane@aist.go.jp

In the original version of the manuscript [1] there was an inadvertent error. The words “25 °C for 48 h” should be replaced with “25 °C for 24 h”. The authors carried out the coating experiments at 25 °C for 1, 3, 6, 12, 24 and 48 h. The apatite coatings formed at 25 °C for 24 and 48 h were found to be identical in physicochemical nature, which was revealed by SEM, EDX, XRD and chemical analysis. Thus, in the animal experiments, the authors used apatite-coated Ti pins fabricated at 25 °C for 24 h. Several corrections are thus required in the abstract, the main text, the figure legends, and the figures (Table 1). The authors would like to apologize for any inconvenience this may have caused to readers of the journal.

The correct versions of Figure 1, Figure 2 and Figure 3 are reprinted below with their legends.

**Table 1:** Corrections in the main text.

Location	Original Version	Correction
Abstract, Page 24366 Line 4	In the present study, an Ap layer was also successfully formed using a one-step method at 25 °C for 48 h in an infusion fluid-based supersaturated calcium phosphate solution, which is clinically useful due to the immersion temperature.	In the present study, an Ap layer was also successfully formed using a one-step method at 25 °C for 24 h in an infusion fluid-based supersaturated calcium phosphate solution, which is clinically useful due to the immersion temperature.
2. Results *2.1. One-Step Formation of Apatite Coating on Ti Pins at 25 °C*; Page 24368 Lines 2–3	In a one-step procedure, increasing the supersaturation of the infusion fluid-based supersaturated CaP solution effectively caused an apatite layer to form on the surface of Ti pins under conditions of 25 °C for 48 h.	In a one-step procedure, increasing the supersaturation of the infusion fluid-based supersaturated CaP solution effectively caused an apatite layer to form on the surface of Ti pins under conditions of 25 °C for 24 h.
Figure legend: Figure 10; Page 24372 Line 2	(a) Averaged values of extraction torque for the UN and Ap groups. Apatite layer was formed at 25 °C for 48 h.	(a) Averaged values of extraction torque for the UN and Ap groups. Apatite layer was formed at 25 °C for 24 h.
3. Discussion; Page 24374 Line 13	An apatite layer was wholly and homogeneously formed on the Ti screw even at room temperature (25 °C) within 48 h without pretreatment of the Ti pin if it is performed in a CaP solution using increased concentrations of calcium and phosphate ions compared with the previous conditions [15,16].	An apatite layer was wholly and homogeneously formed on the Ti screw even at room temperature (25 °C) within 24 h without pretreatment of the Ti pin if it is performed in a CaP solution using increased concentrations of calcium and phosphate ions compared with the previous conditions [15,16].
4. Materials and Methods; *4.2. Immersion of Ti Pins in the Supersaturated CaP Solution*; Page 24375 Line 4	Each Ti pin was immersed in 10 mL of the infusion fluid-based supersaturated CaP solution at 25 °C for 48 h followed by immersion in 2 mL of distilled water for injection (Wasser “Fuso”; Fuso Pharmaceuticals Industries, Osaka, Japan) twice for rinsing.	Each Ti pin was immersed in 10 mL of the infusion fluid-based supersaturated CaP solution at 25 °C for 24 h followed by immersion in 2 mL of distilled water for injection (Wasser “Fuso”; Fuso Pharmaceuticals Industries, Osaka, Japan) twice for rinsing.
5. Conclusions; Page 24377 Line 2	An apatite layer was formed on Ti pins using a clinically useful method: The Ti pins were immersed in an infusion fluid-based supersaturated CaP solution at 25 °C for 48 h.	An apatite layer was formed on Ti pins using a clinically useful method: The Ti pins were immersed in an infusion fluid-based supersaturated CaP solution at 25 °C for 24 h.

**Figure 1:**
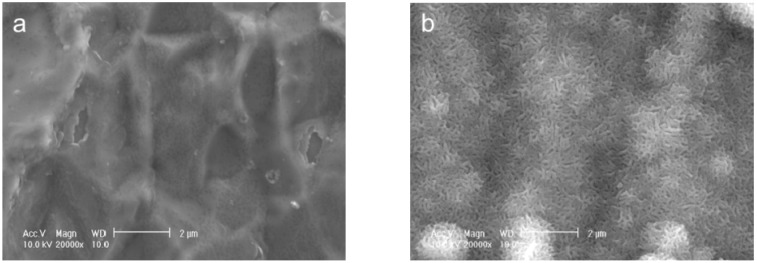
SEM images of the surfaces of Ti pins before (**a**) and after (**b**) immersion in the CaP solution at 25 °C for 24 h.

**Figure 2:**
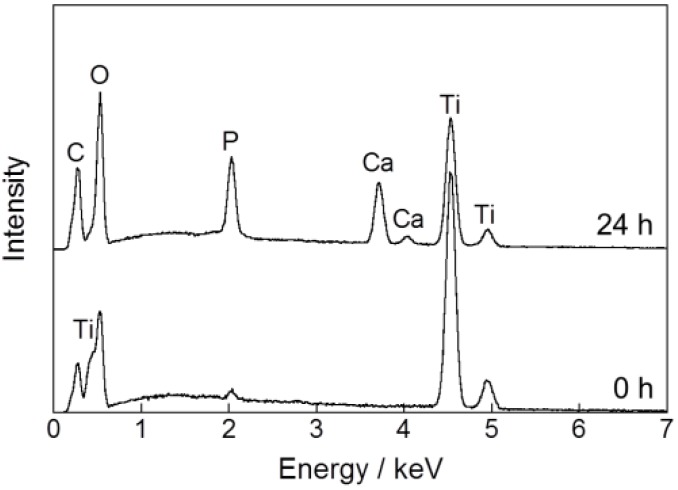
EDX spectra of the surfaces of Ti pins before (0 h) and after (24 h) immersion in the CaP solution at 25 °C for 24 h.

**Figure 3:**
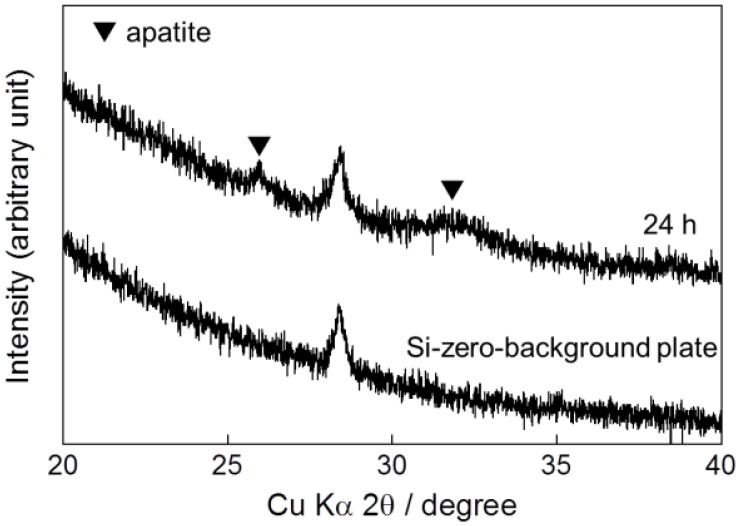
XRD pattern of calcium phosphate deposited on the Ti pin after immersion in the CaP solution at 25 °C for 24 h and that of a silicon-zero-background plate.

The corrected version of the paper can be accessed at http://www.mdpi.com/1422-0067/15/6/9789/s1.

## Supplementary Files

Correction Corrected PDF version (PDF, 5192 KB)

## References

[1] Mutsuzaki H., Sogo Y., Oyane A., Ito A. (2013). Improved bonding of partially osteomyelitic bone to titanium pins owing to biomimetic coating of apatite. Int. J. Mol. Sci..

