# Supplementary material for: Correction: Mutsuzaki, H., et al. Improved Bonding of Partially Osteomyelitic Bone to Titanium Pins Owing to Biomimetic Coating of Apatite. Int. J. Mol. Sci. 2013, 14, 24366–24379
Source: Int J Mol Sci. 2014 May 30;15(6):9789–92. doi: 10.3390/ijms15069789 (PMC4100121; doi:10.3390/ijms15069789)
Supplement: Correction — Corrected PDF version (PDF, 5192 KB) [file ijms-15-09789-s002.pdf]

Int. J. Mol. Sci. **2013**, *14*, 24366-24379; doi:10.3390/ijms141224366

OPEN ACCESS

International Journal of

Molecular Sciences

ISSN 1422-0067

www.mdpi.com/journal/ijms

Article

Improved Bonding of Partially Osteomyelitic Bone to Titanium Pins Owing to Biomimetic Coating of Apatite

Hirotaka Mutsuzaki [1,2], Yu Sogo [2,*], Ayako Oyane [3] and Atsuo Ito [2]

[1] Department of Orthopaedic Surgery, Ibaraki Prefectural University of Health Sciences, 4669-2 Ami Ami-machi, Inashiki-gun, Ibaraki 300-0394, Japan; E-Mail: mutsuzaki@ipu.ac.jp

[2] Human Technology Research Institute, National Institute of Advanced Industrial Science and Technology (AIST), Central 6, 1-1-1, Higashi, Tsukuba-shi, Ibaraki 305-8566, Japan; E-Mail: atsuo-ito@aist.go.jp

[3] Nanosystem Research Institute, National Institute of Advanced Industrial Science and Technology (AIST), Central 4, 1-1-1, Higashi, Tsukuba-shi, Ibaraki 305-8562, Japan; E-Mail: a-oyane@aist.go.jp

* Author to whom correspondence should be addressed: E-Mail: yu-sogou@aist.go.jp; Tel./Fax: +81-29-861-6149.

Received: 16 November 2013; in revised form: 5 December 2013 / Accepted: 11 December 2013 / Published: 13 December 2013

Abstract: Increased fixation strength of the bone-pin interface is important for inhibiting pin loosening after external fixation. In a previous study, an apatite (Ap) layer was formed on anodically oxidized titanium (Ti) pins by immersing them in an infusion fluid-based supersaturated calcium phosphate solution at 37 °C for 48 h. In the present study, an Ap layer was also successfully formed using a one-step method at 25 °C for 24 h in an infusion fluid-based supersaturated calcium phosphate solution, which is clinically useful due to the immersion temperature. After percutaneous implantation in a proximal tibial metaphysis for four weeks in rabbits (n = 20), the Ti pin coated with the Ap layer showed significantly increased extraction torque compared with that of an uncoated Ti screw even with partial osteomyelitis present, owing to dense bone formation on the Ap layer in the cortical and medullary cavity regions. When the infection status was changed from “no osteomyelitis” to “partial osteomyelitis,” the extraction torque in the Ap group with “partial osteomyelitis” was almost identical to that for “no osteomyelitis” cases. These results suggest that the Ap layer formed by the room temperature process could effectively improve the fixation strength of the Ti pin for external fixation clinically even with partial osteomyelitis present.

Keywords: apatite coating; external fixation; fixation strength; osteomyelitis; pin-tract infection

1. Introduction

External fixation is associated with a risk of pin-tract infection in skin and bone tissues, although external fixation is a minimally invasive and useful method for treating bone fractures and deformities [1–7]. Mechanical instability of external fixation pins that leads to loose anchorage at the bone-pin interface can lead to pin-tract infections [8,9]. In the worst-case scenario, chronic osteomyelitis develops because of bacterial infection in a pin tract [8]. A promising strategy for reducing the infection risk is to improve fixation strength at the bone-pin interface, especially for long-term implantation.

Apatite coatings on external fixation pins have improved the fixation strength at the bone-pin interface [10–16]. Plasma-sprayed apatite coating has increased the extraction torque of various external fixation pins by 53%–124% compared with that of uncoated pins [10,11]. Other approaches to form an apatite coating include those using supersaturated calcium phosphate (CaP) solutions [15–21]. Conventional supersaturated solution methods have been generally two-step techniques composed of a physicochemical modification step of substrates and an immersion step in a supersaturated CaP solution prepared from chemical reagents [17–19]. We developed a method wherein an apatite layer was formed on anodically oxidized titanium (Ti) pins just by immersing the pins in an infusion fluid-based supersaturated CaP solution at 37 °C for 48 h [15,16]. This particular solution has the advantage of biological safety over other supersaturated CaP solutions prepared from chemical reagents. The resulting apatite layer on Ti pins led to the formation of dense bone in the cortical and medullary cavity regions after implantation. As a result, the extraction torque values of Ti pins coated with the apatite layer were significantly higher (by 29.9%–46.5%) than those of uncoated pins after percutaneous implantation for four weeks in rabbits [15,16]. However, the extraction torque data used were those for unloosened pins corresponding to "no redness" and "skin infection" cases diagnosed by macroscopic visual inspection. Cases with screw loosening were diagnosed as "osteomyelitis," and their data were excluded from the extraction torque analysis. Hence, the extraction torque information used previously consisted of data for cases of no infection, skin infection without osteomyelitis, and skin infection with osteomyelitis but without screw loosening. Therefore, it is not clear whether apatite-coated Ti pins have an advantage in fixation strength over uncoated Ti pins under different osteomyelitic conditions.

A method that immerses Ti pins at room temperature (25 °C) is clinically more useful than the previous method at 37 °C. Although the room temperature methods for apatite layer formation using infusion fluid-based supersaturated CaP solutions have been reported for Ti rods, they were two-step techniques [20]. The Ti rods required a pretreatment step prior to an immersion step in an infusion fluid-based supersaturated CaP solution [20].

The first purpose of the present study was to develop a one-step immersion method at 25 °C for coating apatite on Ti pins using an infusion fluid-based supersaturated CaP solution. The second purpose was to evaluate the extraction torque of apatite-coated Ti pins in comparison with that of uncoated Ti pins. The special interest here was to clarify the relation between the degree of osteomyelitis and the

extraction torque of apatite-coated and uncoated Ti pins. We therefore assessed the degree of osteomyelitis histologically.

2. Results

2.1. One-Step Formation of Apatite Coating on Ti Pins at 25 °C

In a one-step procedure, increasing the supersaturation of the infusion fluid-based supersaturated CaP solution effectively caused an apatite layer to form on the surface of Ti pins under conditions of 25 °C for 24 h. After immersion in an infusion fluid-based supersaturated CaP solution with 2.4 and 1.6 times higher calcium and phosphate ion concentrations, respectively, than the previous solution, the surfaces of the Ti pins were wholly and homogeneously coated with a fine-structured layer, as confirmed by scanning electron microscopy (SEM) (Figure 1). Ca and P peaks appeared in an energy dispersive electron probe X-ray analysis (EDX) spectrum of the Ti pin after the immersion (Figure 2), proving that the layer was indeed CaP. An amount of CaP sufficient for an X-ray diffraction (XRD) measurement was collected from the Ti pin after the immersion. The XRD profiles showed the peaks corresponding to those for low-crystalline apatite (Figure 3). Note that no obvious diffraction peak was detected in the 2θ (CuKα) range of 3–5°, which corresponds to the main peak region for octaCaP (data not shown). A Ca/P molar ratio of the layer in the range of 1.40–1.44 was revealed using inductively coupled plasma atomic emission spectroscopy (ICP). This finding suggests that the deposited CaP in the layer was not apatite in a strict sense but a mixture or intermediate phase of apatite and its precursors (octaCaP, amorphous CaP), although these precursors were not clearly detected by XRD (Figure 3). Thickness of the calcium phosphate layer was estimated to be 2.9 μm by a CCD laser micrometer.

Figure 1. SEM images of the surfaces of Ti pins before (**a**) and after (**b**) immersion in the CaP solution at 25 °C for 24 h. Scale bar, 2 μm.

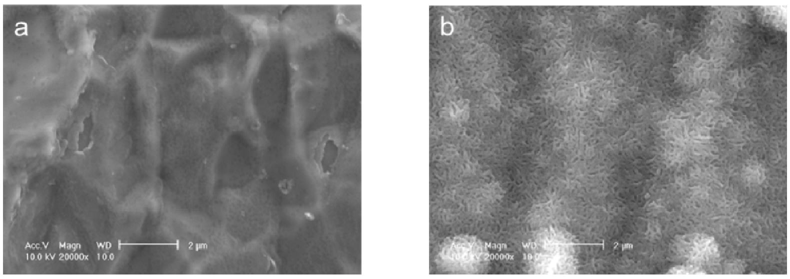

Figure 2. EDX spectra of the surfaces of Ti pins before (0 h) and after (24 h) immersion in the CaP solution at 25 °C for 24 h.

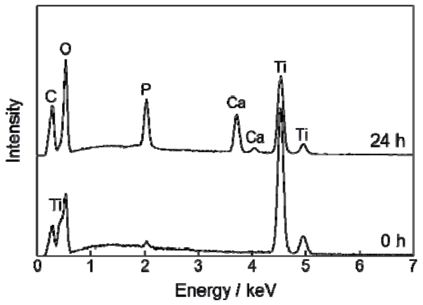

Figure 3. XRD pattern of calcium phosphate deposited on the Ti pin after immersion in the CaP solution at 25 °C for 24 h and that of a silicon-zero-background plate.

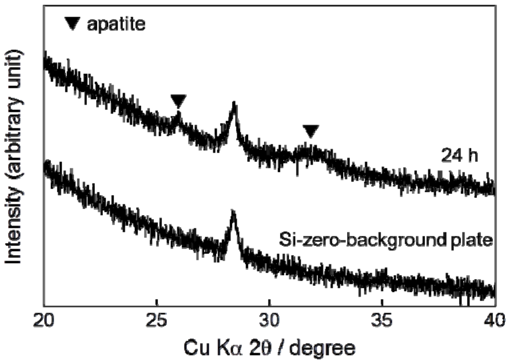

2.2. Classification of Pin-Tract Infections by Visual Inspection

Figure 4 shows typical appearances of Grades 0v, 1v, and 2v after visual inspection of pin-tract infections. The rates of Grade 0v (no redness) for the uncoated Ti pin (UN) and the apatite-coated Ti pin (Ap) groups were 55.0% ($n = 11$) and 40.0% ($n = 8$), respectively. The rates of Grade 1v (skin infection without pin loosening) for the UN and Ap groups were 40.0% ($n = 8$) and 55.0% ($n = 11$), respectively. The rates of Grade 2v (infection with pin loosening) for the UN and Ap groups were 5.0% ($n = 1$) and 5.0% ($n = 1$), respectively. There were no significant differences in the Grade 0v:1v:2v ratio between the UN and Ap groups ($p = 0.3304$).

Figure 4. Typical appearances of pin-tract infection four weeks after the operation. Grade 0v: No redness, discharge, or pin loosening (**a**); Grade 1v: Redness and discharge around the pin but no pin loosening (**b**); Grade 2v: Redness and discharge around the pin, with pin loosening due to osteomyelitis (**c**).

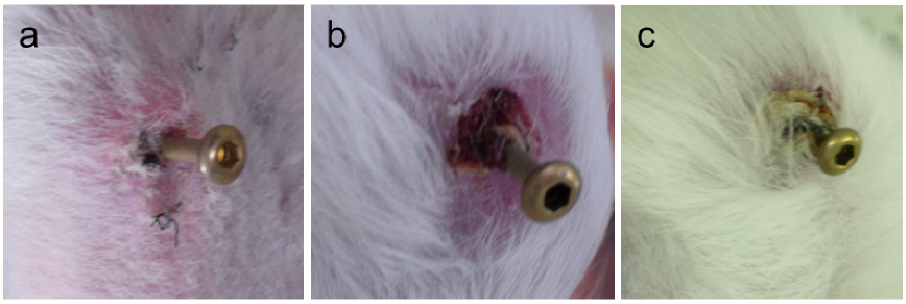

2.3. Classification of Inflammation Grade by Histological Observation

Figure 5 shows typical histological appearances for Grades 0s, 1s, and 2s inflammation in the soft tissue along the pin tract. The rates of Grade 0s (no inflammation) for the UN and Ap groups were 10.0% ($n = 2$) and 10.0% ($n = 2$), respectively. The rates of Grade 1s (partial inflammation) for the UN and Ap groups were 40.0% ($n = 8$) and 35.0% ($n = 7$), respectively. The rates of Grade 2s (severe inflammation) for the UN and Ap groups were 50.0% ($n = 10$) and 55.0% ($n = 11$), respectively. There were no significant differences in the Grade 0s:1s:2s ratio between the UN and Ap groups ($p = 0.9056$).

Figure 5. Typical histological appearances of inflammation in soft tissue along the pin tract four weeks after operation. Grade 0s: No inflammation (**a**); Grade 1s: Partial inflammation (**b**); Grade 2s: Severe inflammation (**c**).

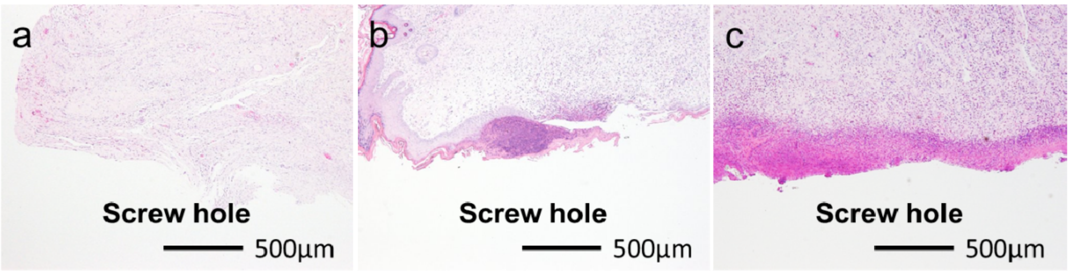

Figure 6 shows typical histological appearances for Grades 0b, 1b, and 2b inflammation in bone tissue along the pin tract. The rates of Grade 0b (no osteomyelitis) for the UN and Ap groups were 75.0% (n = 15) and 50.0% (n = 10), respectively. The rates of Grade 1b (partial osteomyelitis) for the UN and Ap groups were 20.0% (n = 4) and 45.0% (n = 9), respectively. The rates of Grade 2b (severe osteomyelitis) for the UN and Ap groups were 5.0% (n = 1) and 5.0% (n = 1), respectively. There were no significant differences in the Grade 0b:1b:2b ratio between UN and Ap groups (p = 0.0873) (Figure 7). In the UN group, no or very little bone was formed in medullary cavity regions with Grades 0b and 1b osteomyelitis. In the Ap group, however, dense bone was formed in the cortical and medullary cavity regions that displayed not only Grade 0b osteomyelitis but also Grade 1b (Figure 8).

Figure 6. Typical histological appearances of inflammation in bone tissue along the pin tract four weeks after operation. Grade 0b: No osteomyelitis (**a**); Grade 1b: Partial osteomyelitis (**b**); Grade 2b: Severe osteomyelitis (**c**).

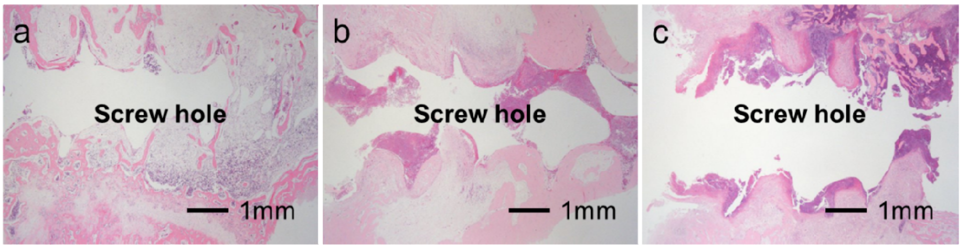

Figure 7. Comparison of differences in osteomyelitis grades classified according to histological observations between the UN and Ap groups. The apatite layer was formed at 25 °C.

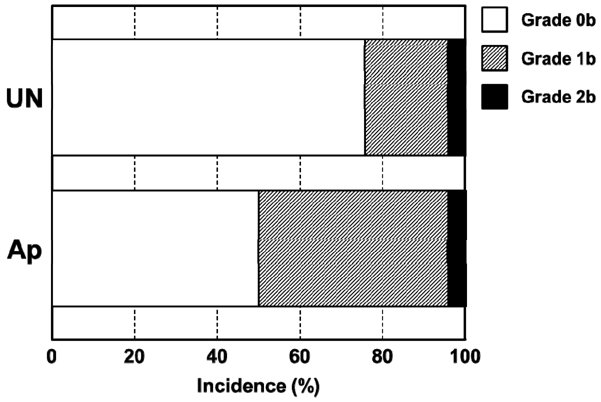

Figure 8. Histological section showing partial osteomyelitis (Grade 1b). (**a**) UN group: No or very little bone was formed in medullary cavity regions (arrows); (**b**) Ap group: Dense bone was formed in the cortical and medullary cavity regions (arrows) even in the presence of partial osteomyelitis.

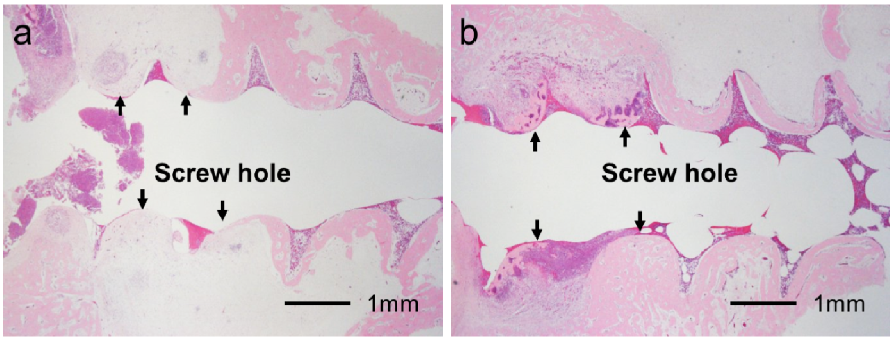

Reanalysis of the rates of Grade 0b, 1b, and 2b osteomyelitis in our previous data obtained using apatite-coated Ti pin prepared at 37 °C for 48 h [16] produced the following results: The rates of Grade 0b osteomyelitis for the UN and Ap groups were 18.75% ($n = 3$) and 37.5% ($n = 6$), respectively. The rate of Grade 1b for the UN and Ap groups were 68.75% ($n = 11$) and 56.25% ($n = 9$), respectively. The rates of Grade 2b for the UN and Ap groups were 12.5% ($n = 2$) and 6.25% ($n = 1$), respectively. There were no significant differences in the Grade 0b:1b:2b ratio between the UN and Ap groups ($p = 0.2800$).

2.4. Bacterial Identification in Pin Tracts

The apatite layer had no effect on resistance to bacterial invasion. According to the bacterial culture results, the detection rates of *Staphylococcus aureus* and *Escherichia coli*, the most typical toxic bacteria, in pin tracts tended to be greater in the Ap group than in the UN group (Table 1). The most frequently detected bacterium in pin tracts of the Ap group was *S. aureus*. Among these two bacterial species, the detection frequency of *S. aureus* was markedly higher in the pin tracts in which infection Grades were classified as 1b and 2b (Figure 9). This result suggested that *S. aureus* was the causative bacterium of osteomyelitis in this study.

Table 1. Detection rates of bacteria in pin tracts for the UN and Ap groups.

Bacteria	UN (%)	Ap (%)
S. aureus	35	65
S. epidermidis	0	5
S. auricularis	60	20
Corynebacterium sp.	20	0
E. coli	5	15
GNF-GNR *	20	5
No bacteria	0	5

* Glucose nonfermentative gram-negative rod.

Figure 9. Detection rates of *S. aureus* and *E. coli* in bone tissue, by inflammation grade.

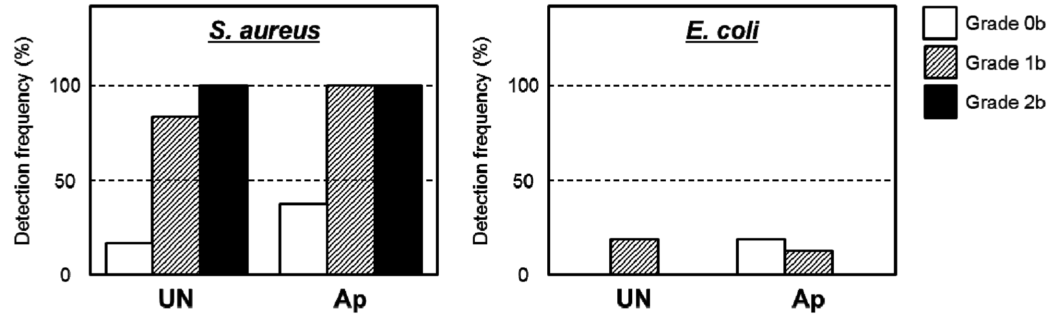

2.5. Extraction Torque

In our previous study, the extraction torque of the Ti pin with an apatite layer formed at 37 °C for 48 h was significantly higher by 46.5% than that of the uncoated Ti pin when cases of Grade 2v osteomyelitis (visual inspection) were excluded [16]. However, it was unclear how effective the apatite layer was in regard to increasing fixation strength in cases of histological Grade 1b or 2b osteomyelitis that could be classified as Grade 0v or 1v visually. In the present study, it was found that the apatite layer has no effect for Grade 2b osteomyelitis (Figure 10a). The averaged value of extraction torque for merged Grades 0b + 1b (no partial osteomyelitis) was significantly higher ($p = 0.0474$) in the Ap group (0.28 ± 0.10 Nm; $n = 19$) than in the UN group (0.23 ± 0.07 Nm; $n = 19$). Similarly, the averaged values of extraction torque for individual Grade 0b and 1b groups in Ap group were higher than those in UN group (p = 0.0701 and 0.0235 for Grade 0b and 1b, respectively) with a significant difference being present only in Grade 1b.

Figure 10. (**a**) Averaged values of extraction torque for the UN and Ap groups. Apatite layer was formed at 25 °C for 24 h. The values were averaged using total data, data for Grades 0b plus 1b, and data only for Grades 0b, 1b, and 2b, respectively; (**b**) Results of the reanalysis of the averaged extraction torque values for the UN and Ap groups [16]. Apatite layer was formed at 37 °C for 48 h. The values are averaged using total data, data for Grades 0b plus 1b, and data only for Grades 0b, 1b, and 2b, respectively.

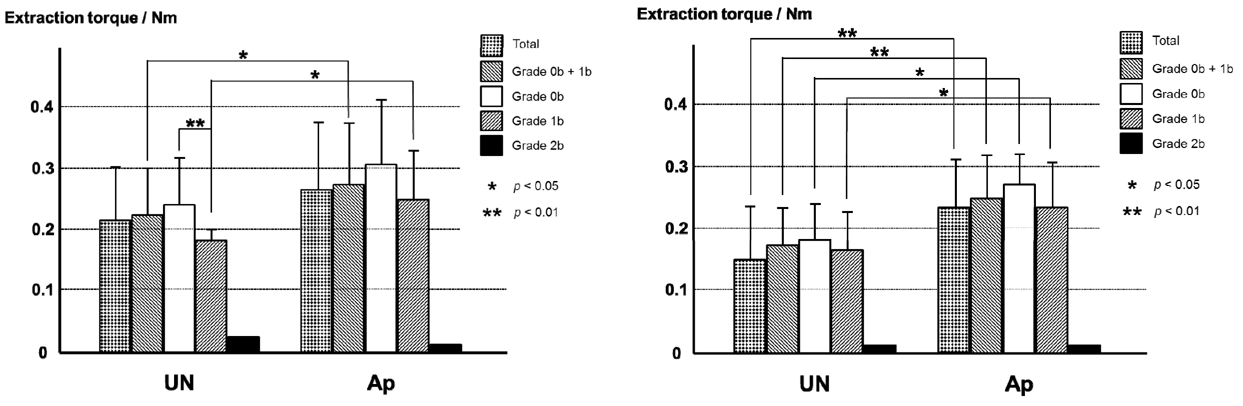

(**a**) (**b**)

When the bone infection status was Grade 0b − 1b, the extraction torque significantly decreased in the UN group ($p = 0.0096$). In contrast, in the Ap group the extraction torque for Grade 0b was almost

identical to that for Grade 1b ($p = 0.1135$). Therefore, in the total specimens, there was no significant difference in the extraction torque between the UN and Ap groups ($p = 0.0772$).

Reanalysis for extraction torque of our previous data obtained using apatite-coated Ti pins prepared at 37 °C for 48 h [16] on the basis of Grades 0b, 1b, and 2b showed a similar tendency (Figure 10b): The apatite layer has no effect on Grade 2b osteomyelitis. The averaged value of extraction torque for the merged Grade 0b + 1b (no partial osteomyelitis) in the Ap group was significantly higher ($p = 0.0012$) than that in UN group. The averaged values of extraction torque for individual Grade 0b and 1b in the Ap group were significantly higher than those in the UN group ($p = 0.0315$) and for Grades 0b and 1b, $p = 0.0184$. When the infection status was impaired at Grades 0b − 1b, the extraction torques were not significantly different in the two groups ($p = 0.3068$ for UN and $p = 0.1689$ for Ap). For the total specimens, there were significant differences in the extraction torque between the UN and Ap groups ($p = 0.0044$).

Extraction torque values for the apatite-coated Ti pins prepared by the one-step method at 25 °C are at the same level as those for the apatite-coated Ti pins prepared by the previous method at 37 °C (Figure 10a,b).

3. Discussion

The most important finding of the present study was that the apatite layer formed by the one-step method at room temperature increased the fixation strength of the Ti screw even in the presence of partial osteomyelitis. The experiment was performed with a four-week percutaneous implantation of the Ti pin in the proximal tibial metaphysis of rabbits. The extraction torque of the uncoated Ti pins classified as Grade 1b (partial osteomyelitis) was lower than that with Grade 0b (no osteomyelitis). On the other hand, in the Ap group, the extraction torque for Grade 1b was almost identical to that for Grade 0b. Furthermore, dense bone formation was observed in the cortical and medullary cavity regions in the Ap group, even in the presence of partial osteomyelitis, whereas no or very little bone formation was observed in the medullary cavity in the UN group.

The reason for the increase in extraction torque in the group with Grade 0b + 1b in the Ap group is that there was dense bone formation in the cortical and medullary cavity regions caused by the osteoconductivity or osteointegrating activity of apatite [10,11,15,16]. Thus, the pin and surrounding bone tissue were likely to integrate when the pin was coated with an apatite layer [15,16,22]. Based on these considerations, even when partial osteomyelitis is clinically observed in the bone-pin interface the apatite-coated Ti screw is likely to retain its bone fixation strength for a longer period than the uncoated Ti screw. This effect should be advantageous for long-term implantation of external fixation, such as for bone transport, treatment of an open fracture, or deformity correction. Plasma-sprayed apatite coatings also increase the extraction torque of various external skeletal fixation pins by 53%–124% compared with that of uncoated pins [10,11]. However, apatite-coated dental implants have an increased risk of bacterial colonization with an increasing ailing period and larger peri-implant defects [23,24]. Therefore, long-term implantation studies of the apatite-coated Ti pin to clarify such an effect on bacterial colonization are necessary.

Despite the improvement in fixation, the apatite layer formed by the 25 °C immersion process showed no infection-reducing effect in either soft or bone tissues. These results were similar to those of

our previous studies [15,16]. In this study, *S. aureus* was detected in the infected pin tract at similar frequencies for both the uncoated and apatite-coated Ti screws. The causal bacterium (*S. aureus*) can be a major problem not only for humans but for animals as well [1–9]. To reduce pin tract infection, antibiotic-containing wound devices and/or fibroblast growth facter-2 (FGF-2) to accelerate wound healing could be necessary [16,20]. It has been reported that FGF-2 can be immobilized within an apatite layer by supplementing a supersaturated CaP solution with FGF-2 in the previous method at 37 °C [16]. The resulting FGF-2-apatite composite layer on the Ti pin reduced pin tract infection rate in the same animal model [16]. When a sponge pad made of poly(ε-caprolactone) containing cefazolin sodium (an antibiotic) was put on the skin around this Ti pin, the infection rate was further reduced [25]. Such approaches would also be effective in reducing infection rate for the apatite-coated Ti pins prepared by the present room temperature method.

An apatite layer was wholly and homogeneously formed on the Ti screw even at room temperature (25 °C) within 24 h without pretreatment of the Ti pin if it is performed in a CaP solution using increased concentrations of calcium and phosphate ions compared with the previous conditions [15,16]. The experimental results suggested that the new CaP solution, with increased calcium and phosphate ion concentrations, is effective in forming a low-crystalline apatite layer even at room temperature. Extraction torque values are the same for the pins prepared by the one-step method at 25 °C and those prepared by the previous method at 37 °C regardless of whether there is Grade 0b osteomyelitis (none) or Grade 1b (partial osteomyelitis).

Based on these facts, the one-step method is more useful than the previous method in terms of the immersion temperature [15,16], with the chemistry and crystallinity of the resulting CaP different from those in the previous method. The difference in chemistry was clear as the molar ratio of the CaP prepared by the one-step method at 25 °C was 1.40–1.44, whereas the previous molar ratio was 1.575 ± 0.005 [15]. A difference in crystallinity would be present because the lower synthetic temperature causes lower crystallinity of apatite [26]. In addition, the layer formed in the one-step method had a submicron-scale porous structure, whereas that formed using the previous method was an aggregate of dense and nano-sized particles [15]. Thus, the lower-temperature process has the advantage of preserving the biological activity of signal molecules if the molecules are intended to be contained in the solution for co-precipitation with apatite [16,19,27].

4. Materials and Methods

4.1. Preparation of an Infusion Fluid-Based Supersaturated CaP Solution

A supersaturated CaP solution was aseptically prepared by mixing five clinically available infusion fluids: Ringer's solution (Ca^{2+} 2.25 mM) (Otsuka Pharmaceuticals, Tokushima, Japan) and calcium chloride corrective injection 1 mEq/mL (Ca^{2+} 500 mM) (Otsuka Pharmaceuticals, Tokushima, Japan) as calcium sources; Klinisalz® (PO_4^{3-} 10 mM) (I'rom Pharmaceuticals, Tokyo, Japan) and dipotassium phosphate corrective injection 1 mEq/mL (PO_4^{3-} 500 mM) (Otsuka Pharmaceuticals, Tokushima, Japan) as phosphorus sources; and Meylon® Injection 7% ($NaHCO_3$ 833 mM) (Otsuka Pharmaceuticals, Tokushima, Japan) as an alkalinizer. The chemical compositions of the CaP solution are summarized in Table 2. The calcium and phosphate ion concentrations in the CaP solution were increased by 2.43 and

1.62 times, respectively, over those in our previous infusion fluid-based CaP solution to maintain a sufficient degree of supersaturation even at 25 °C by compensating for the retrograde solubility of apatite with temperature [15,16,22,28].

Table 2. Chemical composition of the infusion fluid-based supersaturated CaP solutions prepared in the previous and present studies.

Chemical components	Present study (mM)	Previous study (mM) [15]
Na^+	147.23	138.87
K^+	9.92	7.39
Ca^{2+}	8.92	3.67
Mg^{2+}	0.24	0.22
Cl^-	153.46	134.39
$H_2PO_4^-$	2.97	1.83
HCO_3^-	15.09	15.09
CH_3COO^-	1.9	1.8
xylitol	31.65	29.93

4.2. Immersion of Ti Pins in the Supersaturated CaP Solution

The Ti pins used were commercially available, gamma ray-sterilized titanium cancellus screws (#407-030; Synthes, West Chester, PA, USA) with an anodically oxidized surface. They were 4.0 mm diameter and 30 mm length [15,16,25,29]. Each Ti pin was immersed in 10 mL of the infusion fluid-based supersaturated CaP solution at 25 °C for 24 h followed by immersion in 2 mL of distilled water for injection (Wasser "Fuso"; Fuso Pharmaceuticals Industries, Osaka, Japan) twice for rinsing. The rinsed Ti pins were freeze-dried for later characterization of the surface layer. They were used without drying for animal experiments.

4.3. Characterization of the Surface Layer

The surfaces of Ti pins were observed using an SEM (XL30; FEI Company Ltd., Tokyo, Japan) equipped with an EDX (Genesis 2000; EDAX Japan K.K., Tokyo, Japan). The Ti screws were coated with a thin carbon film before observation. To identify the crystalline phase of the surface layer, the layers were scraped off the Ti screw and mounted on a silicon-zero-background plate for analysis using XRD (Rint 2250; Rigaku, Tokyo, Japan).

The amounts of calcium and phosphorus deposited on the Ti screws were determined by chemical analysis. Each Ti screw was immersed in 2 mL of a 10 mM citric acid-sodium citrate buffer (pH 5.43) at 25 °C for more than 3 h to dissolve the surface layer completely. The resulting solutions were analyzed quantitatively for calcium and phosphorus using ICP (SPS7800; Seiko Instruments Inc., Chiba, Japan).

4.4. Animal Experiments

The surgical technique was the same as that described in our previous studies [15,16,25,29]. Ti screws were implanted into 20 skeletally mature male Japanese white rabbits, weighing approximately 3.0 kg. The rabbits were divided into two groups: 10 rabbits in the apatite-coated Ti screw (Ap) group

and the other 10 in the uncoated Ti screw (UN) group. Percutaneous implantation of Ti screws in the rabbits' proximal tibial metaphyses (Figure 1) was carried out following the method described previously [15,16,25,29]. Briefly, small (10 mm) incisions were made in the skin at the medial proximal tibia aseptically after an intravenous injection of barbiturate (40 mg/kg body weight). Then, a hole, 2.5 mm in diameter, was drilled in the tibial metaphysis and with individual taps for each screw. The Ti screws were then manually inserted into these holes. Two Ti screws in the same group were implanted individually in bilateral proximal tibial metaphyses of the rabbit. Hence, the total number of implanted Ti screws was 20 for each group. After implantation, the skin was apposed with two 3–0 nonabsorbable sutures. Postoperatively, each rabbit was allowed free activities in its own cage. The rabbits did not receive any antibiotics or treatment for their wounds. The rabbits did not receive any postoperative medication against pain either. All of the rabbits were sacrificed four weeks after the operation.

All animal experiments and breeding were performed under the conditions approved by the ethics committees of both the University of Tsukuba and the National Institute of Advanced Industrial Science and Technology (AIST). All activities were done in accordance with the National Institutes of Health Guidelines for the Care and Use of Laboratory Animals (http://grants.nih.gov/grants/olaw/Guide-for-the-care-and-use-of-laboratory-animals.pdf).

4.5. Classification of Pin Tract Infections by Visual Inspection

Four weeks after implantation, pin tract infections were evaluated using a modified Checketts classification before sacrifice [16,25]. Grade 0v corresponds to "no redness", in which no redness, discharge, or pin loosening was observed. Grade 1v corresponds to infections only in the soft tissue, characterized by redness and discharge around the pin without pin loosening. Grade 2v corresponds to infections in both soft and bone tissues, characterized by redness and discharge around the pin associated with pin loosening caused by osteomyelitis. A single physician who was blinded to the group identification of pins evaluated the rabbits for pin tract infections. The result was analyzed by χ^2 test for independence. The significance level was set at $p < 0.05$ for each analysis.

4.6. Histological Analysis

After collection of exudate in the pin tracts, the proximal tibial metaphyses were fixed in 10% neutral buffered formalin, decalcified, and embedded in paraffin. The sections were sliced, 5 μm thick, perpendicular to the tibial longitudinal axis and parallel to the hole of the screw. They were stained with hematoxylin-eosin. The specimens were observed histologically using a light microscope (BX-51; Olympus Optical Co., Ltd., Tokyo, Japan) to evaluate the grade of pin-tract inflammation in soft and bone tissues.

For the soft tissue, Grade 0s corresponds to "no inflammation", where no inflammation is observed in the surrounding soft tissue along the whole length of boundary lines between the pin and soft tissue. Grade 2s corresponds to "severe inflammation", where inflammation is observed in the surrounding soft tissue along the whole length of boundary lines between the pin and soft tissue [25,29]. Grade 1s is a status between Grades 0s and 2s and corresponds to "partial inflammation", where inflammation is observed in the surrounding soft tissue along only a part of the length of boundary lines between the pin and soft tissue.

Similarly, for the bone tissue, Grade 0b corresponds to "no osteomyelitis", where no inflammation is observed in the surrounding bone tissue along the whole length of the boundary line between the screw and bone tissue. Grade 2b corresponds to "severe osteomyelitis", where inflammation is observed in the surrounding bone tissue along the whole length of the boundary line between the screw and bone tissue [25]. Grade 1b is a status between Grades 0b and 2b, and corresponds to "partial osteomyelitis", where inflammation is observed in the surrounding bone tissue along only a part of the length of boundary line between the screw and bone tissue.

A single physician who was blinded to the results of the histological examination evaluated the soft and bone tissues for pin-track inflammation. The results were analyzed by χ^2 test for independence. The significance level was set at $p < 0.05$ for each analysis.

4.7. Reanalysis of Osteomyelitis Status and Extraction Torque Data

Reanalysis for osteomyelitis status and extraction data were carried out using previous data [16]. Evaluations of the grade of pin-tract inflammation in the soft and bone tissues were performed using histological sections, as described in Section 4.6. Extraction torque data were analyzed on the basis of the grade of pin-tract inflammation.

4.8. Bacterial Culture and Identification

After complete removal of Ti screws, exudate around each Ti screw was collected with a cotton swab. The swabs with exudate were consigned to a company for clinical laboratory testing (SRL. Inc. Tachikawa, Tokyo, Japan) to detect major bacterial species: *S. aureus* and *E. coli*.

4.9. Biomechanical Analysis

After sacrificing the rabbits, the extraction torque of the Ti screw was measured using a torque-measuring apparatus (HTG2-5N; Imada Co., Ltd., Toyohashi, Japan). The extraction torque data for the Ap and UN groups were compared using Student's t-test at a significance level of $p < 0.05$.

5. Conclusions

An apatite layer was formed on Ti pins using a clinically useful method: The Ti pins were immersed in an infusion fluid-based supersaturated CaP solution at 25 °C for 24 h. Even with partial osteomyelitis present, the apatite-coated Ti pin exhibited a higher extraction torque than uncoated Ti pins after percutaneous implantation in the rabbit proximal tibial metaphysis for four weeks owing to dense bone formation on the apatite layer in the cortical and medullary cavity regions. When the infection status was considered under the conditions of "no osteomyelitis" and "partial osteomyelitis", the extraction torque was almost the same in the Ap group. In addition, extraction torque values were at the same level for pins prepared by the one-step method at 25 °C (introduced herein) and those prepared by the previous method at 37 °C. These results suggest (1) that the apatite layer formed by the one-step method at room temperature is useful, and (2) it effectively improves fixation strength of Ti pins for external fixation clinically, even in the presence of partial osteomyelitis.

Conflicts of Interest

The authors declare no conflict of interest.

References

1. Mahan, J.; Seligson, D.; Henry, S.L.; Hynes, P.: Dobbins, J. Factors in pin tract infections. *Orthopedics* **1991**, *14*, 305–308.
2. Sims, M.; Saleh, M. External fixation—The incidence of pin site infection: Aprospective audit. *J. Orthop. Nurs.* **2000**, *4*, 59–63.
3. Parameswaran, A.D.; Roberts, C.S.; Seligson, D.; Voor, M. Pin tract infection with contemporary external fixation: How much of a problem? *J. Orthop. Trauma* **2003**, *17*, 503–507.
4. Rafique, A.; Ghani, S.; Sadiq, M.; Siddiqui, I.A. Kirschner wire pin tract infection rates between percutaneous and buried wires in treating metacarpal and phalangeal fractures. *J. Coll. Phys. Surg. Pakistan* **2006**, *16*, 518–520.
5. Zlowodzki, M.; Prakash, J.S.; Aggerwal, N.K. External fixation of complex femoral shaft fracture. *Int. Orthop.* **2007**, *31*, 409–413.
6. Schalamon, J.; Petnehazy, T.; Ainoedhofer, H.; Zwick, E.B.; Singer, G.; Hoellwarth, M.E. Pin tract infection with external fixation of pediatric fractures. *J. Pediatr. Surg.* **2007**, *42*, 1584–1587.
7. W-Dahl, A.; Toksvig-Larsen, S. No clinical benefits using a new design of pins for external fixation: A randomized study in 50 patients operated on by the hemicallotasis technique. *Arch. Orthop. Trauma Surg.* **2008**, *128*, 661–667.
8. Green, S.A.; Ripley, M.J. Chronic osteomyelitis in pin tracks. *J. Bone Joint Surg. Am.* **1984**, *66*, 1092–1098.
9. Saithna, A. The influence of hydroxyApatite coating of external fixator pins on pin loosening and pin tract in fection: A systematic review. *Injury* **2010**, *41*, 128–132.
10. Moroni, A.; Heikkila, J.; Magyar, G.; Toksvig-Larsen, S.; Giannini, S. Fixation strength and pin tract infection of hydroxyapatite-coated tapered pins. *Clin. Ortop. Relat. Res.* **2001**, *388*, 209–217.
11. Moroni, A.; Cadossi, M.; Romagnoli, M.; Faldini, C.; Giannini, S. A biomechanical and histological analysis of standard versus hydroxyApatite-coated pins for external fixation. *J. Biomed. Mater. Res. B* **2008**, *86*, 417–421.
12. Pizà, G.; Caja, V.L.; González-Viejo, M.A.; Navarro, A. HydroxyApatite-coated external-fixation pins. The effect on pin loosening and pin-track infection in leg lengthening for short stature. *J. Bone Joint Surg. Br.* **2004**, *86*, 892–897.
13. Placzek, R.; Ruffer, M.; Deuretzbacher, G.; Heijens, E.; Meiss, A.L. The fixation strength of hydroxyApatite-coated Schanz screws and standard stainless steel Schanz screws in lower extremity lengthening: A comparison based on a new torque value index: The fixation index. *Arch. Orthop. Trauma Surg.* **2006**, *126*, 369–373.
14. Pieske, O.; Kaltenhauser, F.; Pichlmaier, L.; Schramm, N., Trentzsch, H., Löffler, T.; Greiner, A.; Piltz, S. Clinical benefit of hydroxyApatite-coated pins compared with stainless steel pins in external fixation at the wrist: A randomised prospective study. *Injury* **2010**, *41*, 1031–1036.

15. Mutsuzaki, H.; Ito, A.; Sakane, M.; Sogo, Y.; Oyane, A.; Ebihara, Y.; Ichinose, N.; Ochiai, N. Calcium phosphate coating formed in infusion fluid mixture to enhance fixation strength of titanium screws. *J. Mater. Sci.* **2007**, *18*, 1799–1808.
16. Mutsuzaki, H.; Ito, A.; Sakane, M.; Sogo, Y.; Oyane, A.; Ochiai, N. FGF-2-apatite composite layers on titanium screws to reduce pin tract infection rate. *J. Biomed. Mater. Res. B* **2008**, *86*, 365–374.
17. Chiono, V.; Gentile, P.; Boccafoschi, F.; Carmagnola, I.; Ninov, M.; Georgieva, V.; Georgiev, G.; Ciardelli, G. Photoactive chitosan switching on bone-like apatite deposition. *Biomacromolecules* **2010**, *11*, 309–315.
18. Oyane, A. Development of apatite-based composites by a biomimetic process for biomedical applications. *J. Ceram. Soc. Jpn.* **2010**,*118*, 77–81.
19. Oyane, A.; Wang, X.P.; Sogo, Y.; Ito, A.; Tsurushima, H. Calcium phosphate composite layers for surface-mediated gene transfer. *Acta Biomater.* **2012**, *8*, 2034–2046.
20. Li, X.; Wang, X.P.; Ito, A.; Sogo, Y.; Cheng, K.; Oyane, A.; Yamazaki, A. Effect of coprecipitation temperature on the properties and activity of fibroblast growth factor-2 apatite composite layer. *Mater. Sci. Eng. C* **2009**, 29, 216–221.
21. Sogo, Y.; Ito, A.; Onoguchi, M.; Oyane, A.; Tsurushima, H.; Ichinose, N. Formation of FGF-2 and calcium phosphate composite layer on hydroxyapatite ceramic for promoting bone formation. *Biomed. Mater.* **2007**, *2*, S175–S180.
22. LeGeros, R.Z. Apatites in biological systems. *Prog. Cryst. Growth Charact.* **1981**, *4*, 1–45.
23. Zablotsky, M.H. Hydroxyapatite coatings in implant dentistry. *Implant Dent.* **1992**, *1*, 253–257.
24. Piattelli, A.; Cosci, F.; Scarano, A.; Trisi, P. Localized chronic suppurative bone infection as a sequel of peri-implantitis in a hydroxyapatite-coated dental implant. *Biomaterials* **1995**, *16*, 917–920.
25. Mutsuzaki, H.; Oyane, A.; Sogo, Y.; Sakane, M.; Ito, A. Cefazolin-containing poly(ε-caprolactone) sponge pad to reduce the pin tract infection rate in rabbits. *Asia Pac. Sports Med. Arthrosc. Rehabil. Technol.* **2013**, in press.
26. Yildirim, O.S.; Aksakal, B.; Celik, H.; Vangolu, Y.; Okur, A. An investigation of the effects of hydroxyapatite coatings on the fixation strength of cortical screws. *Med. Eng. Phys.* **2005**, *27*, 221–228.
27. Savarino, L.; Stea, S.; Granchi, D.; Donati, M.E.; Cervellati, M.; Moroni, A.; Paganetto, G.; Pizzoferrato, A. X-ray diffraction of bone at the interface with hydroxyApatite-coated versus uncoated metal implants. *J. Mater. Sci.* **1998**, *9*, 109–115.
28. Elliot, J.C. Hydroxyapatite and Nonstoichiometric Apatites. *Structure and Chemistry of the Apatites and Other Calcium Orthophosphates. Studies in Inorganic Chemistry 18*, 2nd Ed.; Elsevier: Amsterdam, The Netherlands, 1994; Volume 18, pp. 111–190.
29. Mutsuzaki, H.; Ito, A.; Sogo, Y.; Sakane, M.; Oyane, A.; Ochiai, N. Enhanced wound healing associated with Sharpey's fiber-like tissue formation around FGF-2-apatite composite layers on percutaneous titanium screws in rabbits. *Arch. Orthop. Trauma Surg.* **2012**, *132*, 113–121.

© 2013 by the authors; licensee MDPI, Basel, Switzerland. This article is an open access article distributed under the terms and conditions of the Creative Commons Attribution license (http://creativecommons.org/licenses/by/3.0/).
